# Genetic Heterogeneity of Breast Cancer Metastasis May Be Related to miR-21 Regulation of TIMP-3 in Translation

**DOI:** 10.1155/2013/875078

**Published:** 2013-07-10

**Authors:** Jianyi Li, Yang Zhang, Wenhai Zhang, Shi Jia, Rui Tian, Ye Kang, Yan Ma, Dan Li

**Affiliations:** ^1^Department of Breast Surgery, Shengjing Hospital of China Medical University, Shenyang, Liaoning 110004, China; ^2^Ultrasound Diagnosis Center, Shengjing Hospital of China Medical University, Shenyang, Liaoning 110004, China; ^3^Pathological Diagnosis Center, Shengjing Hospital of China Medical University, Shenyang, Liaoning 110004, China

## Abstract

*Purpose.* MicroRNAs are noncoding RNA molecules that posttranscriptionally regulated expression of target gene and implicate the progress of cancer proliferation, differentiation, and apoptosis. The aim of this study is to determine whether microRNA-21 (miR-21), a specific microRNA implicated in multiple aspects of carcinogenesis, promoted breast cancer metastasis by regulating the tissue inhibitor of metalloproteinase 3 (TIMP-3) gene. *Methods.* miR-21 of serum and tissue from 40 patients (30 patients with breast cancer) were detected by real-time quantitative reverse transcriptase polymerase chain reaction (RT-qPCR). TIMP-3 of tissue from the patient was tested by real-time RT-qPCR. Protein expression of TIMP-3 was evaluated by western blotting. Correlation analysis was performed between miR-21 and TIMP-3. *Results.* Of the 40 samples from tissue and serum analyzed, the miR-21 expression was significantly higher in high invasion metastasis group (HIMG) that in low invasion metastasis group (LIMG); the latter was higher than that in normal group (NG). Additionally, the TIMP-3 expression was significantly lower in HIMG than in LIMG; the latter was lower than that in NG. There was significantly inverse correlation between miR-21 and TIMP-3 extracted from tissue. *Conclusion.* Our data suggest that miR-21 could promote metastasis in breast cancer via the regulation of TIMP3 translation, and there was consistency between miR-21 of serum and miR-21 in tissue.

## 1. Introduction

Metastasis is the main reason which cause the treatment failure and death in patients with breast cancer [1]. In clinical work, even in the same pathological type, histological grade, clinical stages and molecular typing, differences between the metastatic probability in patients are huge [2]. In fact, tumor metastasis is still poorly understood for researchers, and deconstruction of genetic heterogeneity is the right way. According to findings previously, endogenous inhibitors of matrix metalloproteinases (MMPs) play an important role in extracellular matrix (ECM) homeostasis and deregulate ECM remodeling which contributes to cancer metastasis [3, 4]. Tissue inhibitor of metalloproteinase (TIMP) balanced the role of MMPs involved in organizing remodeling, thus having an impact on cancer metastasis [5]. On the other hand, the discovery of microRNA regulation of tumor metastasis was considered to be the molecular basis of the genetic heterogeneity of mechanism's important part [6]. Specifically, miR-21 is overexpressed in diverse types of malignancy [7]. Further, recent experiments suggest that miR-21 can regulate the expression of tissue inhibitor of metalloproteinase-3 (TIMP-3) to control the invasion of breast cancer [8]. We sought to determine the role of miR-21 in breast cancer metastasis and to identify whether miR-21-mediated metastasis might be regulated via TIMP-3.

## 2. Methods

### 2.1. Patients and Groups

Human tissue and serum samples were obtained by surgical resection and blood drawing from patients who have been treated in Shengjing Hospital of China Medical University from 2009 to 2010. Inclusion criteria included invasive ductal carcinoma, receiving no neoadjuvant therapy, no history of radiotherapy before, and no previous history of cancer, and no vice-breast cancer. In those patients, there are fifteen persons who entirely meet the following requirements entered the HIMG: tumor diameter less than 2 cm; lymph node metastases; historical grade III; Her2 positive; vascular thrombosis positive; estrogen and progesterone receptor negative; P53 positive; Ki67 positive more than or equal to 14%. There are fifteen persons who completely meet the following requirements entered the LIMG: tumor diameter more than 3 cm; no lymph node metastases and micrometastases; historical grade I; Her2 negative; vascular thrombosis negative; estrogen and progesterone receptor positive; P53 negative; Ki67 positive less than 14% (Table 1). And we choose 10 patients with benign tumor as the control group during the same period (Table 2). By the way, all patients on admission signed complete informed consent.

### 2.2. Serum and Tissue Samples

The preoperative blood was collected and centrifuged, and volume of 2 mL of serum was kept as above. The samples including serum, tumor tissue and normal breast tissue, were preserved temporarily in liquid nitrogen for 30 min following isolated and for long time in deep freezer at −86°C. 

### 2.3. Micrometastasis Detection

If no carcinoma cells were detected in the nodes, immunohistochemistry with cytokeratin antibody CK-22 (Santa Cruz, USA), using a standard immunoperoxidase method (ABC Elite kit, Vector Laboratories, USA), was performed. Micrometastasis was defined as tumor of the size exceeding 0.2 mm and less than or equal to 2 mm in diameter, according to the American Joint Committee of Cancer (AJCC) 7th classification. Hence, isolated tumor cells or tumor cell clusters measuring less than or equal to 0.2 mm in diameter did not meet the definition of micrometastases [9]. Therefore, the patients with such clusters were considered as micro-metastasis negative. All the analysis above was performed by a pathologist from the Breast Group of Pathology Diagnosis Center of our institute.

### 2.4. Real-Time RT-qPCR

Small RNA of serum was isolated by mirVana PARIS Kit (AM1556, ABI, USA). Small RNA and total RNA of breast tissue were extracted by mirVana miRNA Isolation Kit (AM1560, ABI, USA). Reverse transcription was performed with PrimeScript RT reagent kit (DRR037A, Takara, Japan) in a final volume of 10 *μ*L containing RNA 200 ng and other elements followed instruction of protocol. Small RNA was added poly-A tail by poly-A polymerase (NEB, M0276) before reverse transcription using primers in Table 3 as before (Table 3). Real-time quantitative PCR was performed on Roche LightCycler 2.0 with SYBR Premix Ex Taq (DRR041A, Takara, Japan). For each sample, real time PCR was performed in a final volume of 10 *μ*L containing PCR master mix, 50 ng of genomic DNA or 5 ng of cDNA, and primers (250 nM). For negative control, template was replaced by purified non-reverse-transcripted RNA. Each experiment was done in triplicate. Averaged Ct values of GAPDH were subtracted from each averaged interest Ct to give ΔCt.

### 2.5. Western Blot

Protein extracts, SDS-PAGE, electro-transfer, and immunoblotting were following the standard procedure. The TIMP3 expression can be detected by sc-6836 (Santa Cruz, USA), which was against the C-terminal of TIMP3. Internal controls were checked by antibody of GAPDH (KC-5G4, Kangchen Biotech, China). Densitometric analysis was performed using Quantity One (version 4.5, Bio-Rad, USA).

### 2.6. Statistical Analysis

All the data were performed by normality test: the normally distributed data was compared using *t*-test; other data were log-transforming to meet normally distributed and abnormally distributed using Mann-Whitney *U* test. Multiple groups were compared using ANOVA analysis, between the two groups using SNK test (Student-Newman-Keuls), and correlation analysis using Pearson test. *P* < 0.05 was defined as being significant. Statistical analysis was performed using the SPSS software (version 17.0, IBM, USA).

## 3. Result

### 3.1. miR-21 Is Overexpressed in HIMG (Realtime RT-qPCR)

The relative content of miR-21 extracted from tissue in HIMG was 9.34 ± 1.87, LIMG was 4.65 ± 1.44, and NG was 0.00 ± 2.59. There was significant difference in the three groups (*F* = 70.91, *P* < 0.05) (Table 4). The tissue miR-21 expression was significantly higher in HIMG than in LIMG; the latter was higher than that in NG by SNK test (*P* < 0.05) (Figure 2(a)). The relative content of miR-21 extracted from serum in HIMG was 10.91 ± 1.82, LIMG was 7.25 ± 1.49, and NG was 0.00 ± 2.94. There was marked diffidence in the three groups (*F* = 85.38, *P* < 0.05) (Table 4). The serum miR-21 expression was significantly higher in HIMG than LIMG, the latter was higher than NG by SNK test (*P* < 0.05) (Figure 2(b)). The relative content of miR-21 in tissue positively correlates with that in corresponding serum, and the Pearson coefficient was 0.866 (*P* < 0.05) (Table 5, Figure 3(a)).

### 3.2. Protein and mRNA of TIMP-3 Was Contradictory-Expressed in HIMG, LIMG, and NG (Western Blot and Real-Time RT-qPCR)

**Figure 1:**
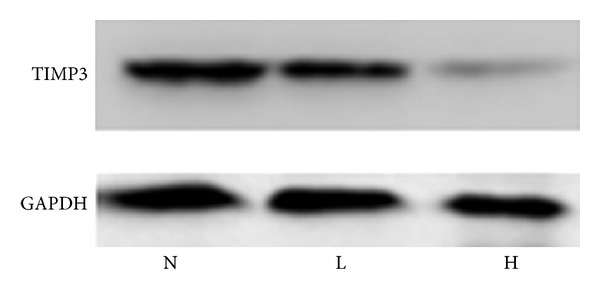
Western blot electrophoresis results, N: normal group, L: low invasion and metastasis group, H: high invasion and metastasis group; it can be seen that TIMP3 protein expression was significantly decreased in high invasion and metastasis group.

**Figure 2:**
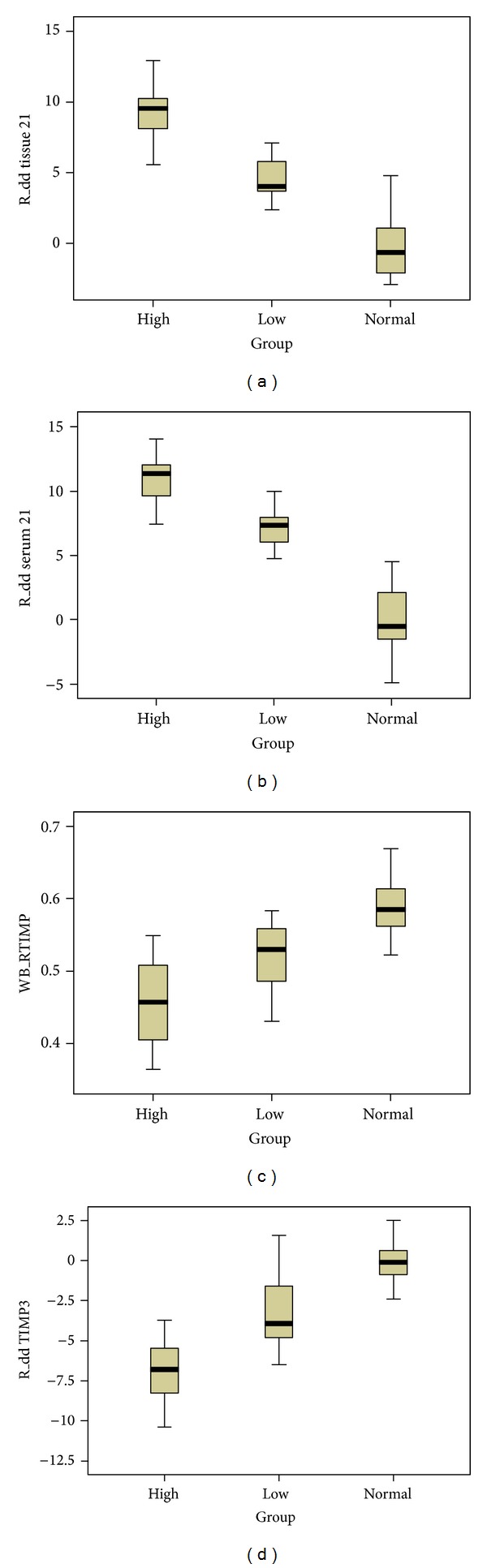
(a) miR-21 in various clinical invasions in breast cancer tissue of the different transcripts. Experimental method: realtime RT-PCR; statistical method: ANOVA showed *P* < 0.05; comparison between groups *P* < 0.05, that is, HIMG > LIMG > NG. (b) miR-21 in various clinical invasions in breast cancer patients serum of the different levels. Experimental method: realtime RT-PCR; statistical method: ANOVA showed *P* < 0.05; comparison between groups *P* < 0.05, that is, HIMG > LIMG > NG. (c) TIMP3 in various clinical invasions in breast tissue of different expressions. Experimental methods: western blot; statistical method: ANOVA showed *P* < 0.05; comparison between groups *P* <0.05, HIMG < LIMG < NG. (d) TIMP3 in various *t* clinical invasions in breast cancer tissue of the different transcripts. Experimental method: realtime RT-PCR; statistical method: ANOVA showed *P* < 0.05; comparison between groups *P* < 0.05, that is, HIMG < LIMG < NG.

The content of TIMP-3 in HIMG was 0.455 ± 0.062, LIMG was 0.517 ± 0.050, and NG was 0.592 ± 0.046. There was striking difference in the three groups (*F* = 19.43, *P* < 0.05) (Table 4). The TIMP-3 protein expression was apparently lower in HIMG than in LIMG, the latter was lower than that in NG by SNK test (*P* < 0.05) (Figures 1 and 2(c)). The relative content of TIMP-3 mRNA in HIMG was −6.90 ± 2.09, LIMG was −3.21 ± 2.25, and NG was 0.00 ± 1.55. There was remarkable difference in the three groups (*F* = 35.28, *P* < 0.05). The TIMP-3 mRNA expression was apparently lower in HIMG than in LIMG; the latter was lower than that in NG by SNK test (*P* < 0.05) (Figure 2(d)).

### 3.3. TIMP-3 Expression Inversely Correlates with miR-21 Relative Content in Breast Tissue

**Figure 3:**
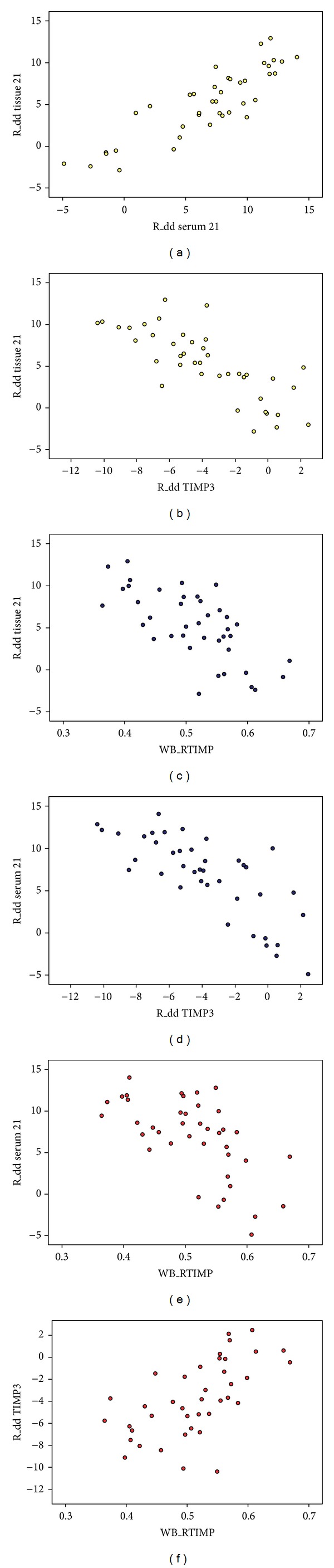
(a) miR-21 transcription in volume of breast tissue and corresponding serum levels in patients with significant correlation, Pearson correlation (positive correlation, correlation coefficient = 0.866, *P* < 0.05). (b) Different clinical invasions of breast tissue in the transcription miR-21 and TIMP3 were significantly correlated (negative correlation, correlation coefficient = −0.778, *P* < 0.05). (c) Different clinical invasion of breast tissue in volume of miR-21 transcription and TIMP3 protein expression was significantly correlated (negative correlation, correlation coefficient = −0.692, *P* < 0.05). (d) miR-21 serum levels and breast tissue volume of TIMP transcription were significantly correlated (negative correlation, correlation coefficient = −0.762, *P* < 0.05). (e) miR-21 serum levels and TIMP breast tissue levels of protein expressions were significantly correlated (negative correlation, correlation coefficient = −0.625, *P* < 0.05). (f) Different clinical invasions of breast tissue levels of TIMP3 transcription and protein expression were also correlated (positive correlation, correlation coefficient = 0.616, *P* < 0.05).

In HIMG with high relative miR-21 expression extracted from tissue, low dose mRNA and protein of TIMP-3 were observed, whereas LIMG with low relative miR-21 expression displayed relatively high amount of TIMP-3 (mRNA and protein), resulting in an apparently inverse correlation between tissue miR-21 expression and TIMP-3 content (Pearson correlation, *r* = −0.778 and −0.692, resp., *P* < 0.05) (Table 5, Figures 3(b) and 3(c)). In HIMG with high relative miR-21 expression extracted from serum, low amounts mRNA and protein of TIMP-3 were observed, whereas LIMG with low relative miR-21 expression displayed relatively high amounts of TIMP-3 (mRNA and protein), resulting in a significantly inverse correlation between serum miR-21 expression and TIMP-3 content (Pearson correlation, *r* = −0.762 and −0.625, resp., *P* < 0.05) (Table 5, Figures 3(d) and 3(e)). There was significant positive correlation between mRNA and protein of TIMP-3 extracted from tissue (Pearson correlation, *r* = 0.616; *P* < 0.05) (Table 5, Figure 3(f)).

## 4. Discussion

**Figure 4:**
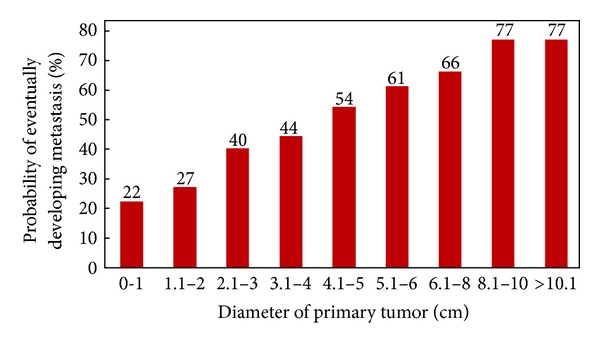
From Heimann and Hellman [10].

Recent experiments in vitro have suggested that degree of degradation of ECM was determined by the balance between MMPs and TIMP, which affected the epithelial mesenchymal transformation (EMT) [11]. EMT is considered to be the initial stage of cancer invasion and metastasis of critical process [12]. The same tumor can be significantly different in prognosis caused by different individuals. This is very important in breast cancer patients, because metastasis is the main reason which causes death. The following indicators can predict the metastasis in various degrees, such as historical grading; tumor thrombus; lymph nodes metastasis and micro-metastasis; estrogen receptor and progesterone receptor; human epithelial growth factors receptor-2; P53 and Ki67 [13–16]. Therefore, those generally accepted indicators were grouped criteria through determining the heterogeneity of clinical breast cancer metastasis difference (Table 1). According to Heimann and Hellman's study in 1998, the probability of breast cancer metastasis increased with the tumor diameter [10]. While we can still find that 22% breast cancer patients whose tumor diameter less than 1 cm had metastasis, 23% had no metastasis more than 10 cm (Figure 4). If the increasing rate in tumor volume was certain, it could make larger tumors have no metastasis or the ability of metastasis even worse, and vice versa. In order to compare the ability of metastatic difference better, the aforementioned view was seen as one of grouping criteria in the study (Table 1). It was the aim of grouping criteria to make the genetic heterogeneity become the main reason which cause the metastasis other than the clinical stage. In the present study, we identified increased expression of miR-21, as compared to LIMG (Table 4). These data were consistent with reports indicating that miR-21 expression increased with miR-21 expression was increasing the progression of clinical stage and shortening survival of patients [17]. In fact, the miR-21 gene is located on chromosome 17q23.2, which is located within the common fragile site FRA17B [18]. This region is frequently found amplified in breast, colon, and lung cancer, consistent with the fact that miR-21 overexpression is widespread in many types of cancer, including the breast [19]. Despite the link of miR-21 to carcinogenesis, little was known regarding the specific mechanism of how miR-21 made cancer progression. Several findings suggested that miR-21 could be impacting matrix metalloproteinases inhibitors, such as TIMP3, that played a crucial role in cancer invasion and metastasis including recent studies that identified TIMP3 as a functional target of miR-21 in cell invasion and metastasis in glioma and cholangiocarcinoma [20, 21]. Recently, Song et al. have proved that miR-21 can regulate the expression of TIMP-3 to control the invasion of breast cancer cell [8]. Our finding reported that microRNA-21 negatively regulated TIMP3 in breast cancer and suggested that TIMP3 might be negatively regulated by miR-21 at the translated level (Table 5). These compelling data supported miR-21 regulation of TIMP3 expression as a novel mechanism impacting genetic heterogeneity of breast cancer invasion and metastasis. But the regulation mechanism needs to be further confirmed by the vitro experiments. Our experiment also demonstrated that the miR-21 in tissue and the miR-21 in serum had a high degree of consistency. Recent study prompted that high circulating miR-21 concentrations correlated significantly with visceral metastasis in patients with breast cancer [22]. This suggested that miR-21 is a predictor of breast cancer metastasis marker with the qualifications.

## Figures and Tables

**Table 1:** 

Grouped criteria	High invasion and metastasis group HIMG	Low invasion and metastasis group LIMG
Diameter by ultrasound (cm)	<2 cm	≧3 cm
Lymph nodes metastasis by HE	Yes	No
Micrometastasis by CK-22	Unnecessary	No
Histological grading	III	I
Tumor embolus	Positive	Negative
Her2 receptor status	Positive	Negative
ER & PR	Negative	Positive
P53	Positive	Negative
Ki67	≧14%	<14%

All indicators of immunohistochemical staining need to meet verification of two pathological diagnosis centers.

**Table 2:** 

Parameters	HIMG	LIMG	Normal group
Age (years)			
Median	42	48	45
Range	(33~60)	(36~64)	(35~62)
Quadrant			
Areolar	3	2	2
Outer upper	5	6	3
Outer lower	4	5	3
Inner lower	1	1	1
Inner upper	2	1	1
Operation			
Mastectomy	12	10	
Tumorectomy	3	5	

**Table 3:** Primers.

Universal reverse transcription primer	GCTGTCAACGATACGCTACGTAACGGCATGACAGTG(TT⋯TT)_24_N(A, G, C)	
U6	F: CTCGCTTCGGCAGCACA	R: AACGCTTCACGAATTTGCGT
miR-21	F: AGCTTATCAGACTGATGTTG	R: GCTGTCAACGATACGCTACGTAACG
TIMP3	F: CTTCCAAGAACGAGTGTCT	R: GGTCTGTGGCATTGATGA
GAPDH	F: GGTGAAGGTCGGAGTCAACG	R: CCATGTAGTTGAGGTCAATGAAG

**Table 4:** 

Sample	Group	Mean ± SD	*F*	*P*
Tissue miR-21	High	9.34 ± 1.87	70.91	0.000
	Low	4.65 ± 1.44		
	Normal	0.00 ± 2.59		

Serum miR-21	High	10.91 ± 1.82	85.38	0.000
	Low	7.25 ± 1.49		
	Normal	0.00 ± 2.94		

TIMP-3 mRNA	High	−6.90 ± 2.09	35.28	0.000
	Low	−3.21 ± 2.25		
	Normal	0.00 ± 1.55		

TIMP-3 protein	High	0.455 ± 0.062	19.43	0.000
	Low	0.517 ± 0.050		
	Normal	0.592 ± 0.046		

Averaged Ct value of normal samples was chosen as reference (ΔΔCt = 0, relative fold increase, RFI = 1). ΔΔCt was calculated by ΔCt subtracted with this reference. Our datum of relative fold index (RFI = 2 − ΔΔCt) obeyed skewed distribution, so we transformed our datum with log2(RFI) to normal distribution. miR-21 expression of tumor tissue and serum samples in the normal group, high and low invasive group has statistical differences and the comparison between each group is statistically significant (*P* < 0.01).

Tumor tissue TIMP-3 mRNA and protein expression levels in each group has statistical differences and the comparison between each group is statistically significant (*P* < 0.01).

**Table 5:** 

Data A	Data B	Correlation coefficient	Sig.
Tissue miR-21	Serum miR-21	0.866	0.000
Tissue miR-21	TIMP-3 mRNA	−0.778	0.000
Tissue miR-21	TIMP-3 Proteins	−0.692	0.000
Serum miR-21	TIMP-3 mRNA	−0.762	0.000
Serum miR-21	TIMP-3 Proteins	−0.625	0.000
TIMP-3 mRNA	TIMP-3 Proteins	0.616	0.000

Each of the tumor tissue and serum in the miR-21, tumor tissue miR-21 and TIMP-3 of the mRNA, tumor tissue miR-21 and TIMP-3 protein, serum miR-21 and tumor tissue of TIMP-3 mRNA, the miR-21 in serum and tumor tissue TIMP-3 protein, and tumor tissue TIMP-3 mRNA and protein do Pearson correlation analysis; correlation coefficients were 0.866, −0.778, −0.692, −0.762, −0.625, and 0.616, with statistical significance.
